# Supplementary material for: Genome-Wide Identification and Expression Analysis of the Strawberry FvbZIP Gene Family and the Role of Key Gene FabZIP46 in Fruit Resistance to Gray Mold
Source: Plants (Basel). 2020 Sep 14;9(9):1199. doi: 10.3390/plants9091199 (PMC7569810; doi:10.3390/plants9091199)
Supplement: Supplementary file 1 [file plants-09-01199-s001.zip › plants-914991-SI/supplementary files/Table S1.docx]

**Table S1 Related information of strawberry bZIP gene family**

| **Gene number** | **Gene accession No.** | **Chromosome** | **Group** | **Isoelectric point** | **Protein length** | **Molecular weight (KDa)** | **Subcellular localization** | **Locus** |
| --- | --- | --- | --- | --- | --- | --- | --- | --- |
| *FvbZIP1* | LOC101293727 | 1 | A | 7.87 | 227 | 25.47 | Nucleus | 8266977 8268513 |
| *FvbZIP2* | LOC101315273 | 1 | S | 4.68 | 157 | 17.85 | Nucleus | 9034546 9036278 |
| *FvbZIP3* | LOC101307640 | 2 | UC | 4.67 | 221 | 25.01 | Nucleus | 333803 338024 |
| *FvbZIP4* | LOC101313165 | 2 | S | 6.79 | 159 | 17.98 | Nucleus | 775925 776937 |
| *FvbZIP5* | LOC101314521 | 2 | F | 6.39 | 276 | 30.04 | Nucleus | 5165864 5169968 |
| *FvbZIP6* | LOC101297199 | 2 | C | 6.4 | 420 | 45.83 | Nucleus | 5515381 5517834 |
| *FvbZIP7* | LOC101303379 | 2 | I | 6.31 | 585 | 64.35 | Nucleus | 11338484 11343025 |
| *FvbZIP8* | LOC101293647 | 2 | A | 6.58 | 310 | 33.77 | Nucleus | 14181286 14186979 |
| *FvbZIP9* | LOC101315311 | 2 | I | 7.19 | 336 | 37.05 | Nucleus | 14582184 14585469 |
| *FvbZIP10* | LOC101298971 | 2 | S | 8.9 | 158 | 18.18 | Nucleus | 20080206 20081125 |
| *FvbZIP11* | LOC101302803 | 2 | A | 10.21 | 448 | 47.87 | Nucleus | 21049954 21053759 |
| *FvbZIP12* | LOC101302614 | 2 | C | 5.21 | 335 | 36.2 | Nucleus | 22175617 22178546 |
| *FvbZIP13* | LOC101300210 | 2 | A | 10 | 216 | 23.38 | Nucleus | 23097235 23098899 |
| *FvbZIP14* | LOC101292368 | 2 | I | 6.93 | 552 | 60.11 | Nucleus | 23513019 23516830 |
| *FvbZIP15* | LOC101314818 | 2 | H | 10.28 | 166 | 18.04 | Nucleus | 24251575 24254480 |
| *FvbZIP16* | LOC101309994 | 2 | D | 7.12 | 545 | 60.31 | Nucleus | 26922767 26929489 |
| *FvbZIP17* | LOC101305244 | 2 | A | 5.36 | 370 | 40.55 | Nucleus | 26941659 26950965 |
| *FvbZIP18* | LOC105350147 | 2 | A | 10.61 | 140 | 16.25 | Nucleus | 26952316 26957776 |
| *FvbZIP19* | LOC101314152 | 2 | S | 7.09 | 171 | 19.15 | Nucleus | 32244987 32246016 |
| *FvbZIP20* | LOC105350403 | 3 | A | 10.14 | 212 | 24.21 | Nucleus | 8606927 8608247 |
| *FvbZIP21* | LOC105350508 | 3 | UC | 6.82 | 343 | 38.23 | Nucleus | 13297705 13300376 |
| *FvbZIP22* | LOC105350507 | 3 | UC | 7.57 | 298 | 33.11 | Nucleus | 13293262 13297458 |
| *FvbZIP23* | LOC101302546 | 3 | I | 6.85 | 434 | 47.13 | Nucleus | 20569002 20571857 |
| *FvbZIP24* | LOC101291997 | 3 | A | 10.16 | 432 | 47 | Nucleus | 21394232 21398130 |
| *FvbZIP25* | LOC101297428 | 4 | H | 10.04 | 173 | 19.76 | Nucleus | 14859686 14861541 |
| *FvbZIP26* | LOC101301954 | 4 | D | 6.51 | 453 | 50.54 | Nucleus | 16054947 16067478 |
| *FvbZIP27* | LOC101293580 | 4 | S | 5.55 | 197 | 22.59 | Nucleus | 16741826 16743118 |
| *FvbZIP28* | LOC101302650 | 5 | G | 4.53 | 262 | 28.7 | Nucleus | 2252582 2255240 |
| *FvbZIP29* | LOC101303420 | 5 | G | 6.03 | 148 | 16.55 | Nucleus | 2268184 2270314 |
| *FvbZIP30* | LOC101299380 | 5 | B | 6.71 | 711 | 76.02 | Nucleus | 3775603 3778161 |
| *FvbZIP31* | LOC101291730 | 5 | S | 9.49 | 114 | 13.51 | Nucleus | 7313237 7313581 |
| *FvbZIP32* | LOC101299962 | 5 | E | 7.99 | 378 | 42.57 | Nucleus | 15959090 15964509 |
| *FvbZIP33* | LOC101301305 | 5 | A | 6.64 | 277 | 29.89 | Nucleus | 17169542 17172430 |
| *FvbZIP34* | LOC101310028 | 5 | H | 4.48 | 320 | 35.01 | Nucleus | 18884381 18885405 |
| *FvbZIP35* | LOC101292506 | 5 | G | 6.86 | 411 | 43.55 | Nucleus | 20506518 20513568 |
| *FvbZIP36* | LOC101299007 | 5 | S | 6.45 | 148 | 17.17 | Nucleus | 24791957 24793729 |
| *FvbZIP37* | LOC101299194 | 5 | G | 6.61 | 343 | 35.8 | Nucleus | 26803089 26806871 |
| *FvbZIP38* | LOC101307125 | 5 | A | 6.76 | 336 | 37.66 | Nucleus | 28016285 28018785 |
| *FvbZIP39* | LOC101292708 | 5 | D | 6.53 | 369 | 41.74 | Nucleus | 28031619 28035422 |
| *FvbZIP40* | LOC101304589 | 6 | C | 4.96 | 426 | 46.75 | Nucleus | 528802 531985 |
| *FvbZIP41* | LOC101305275 | 6 | D | 6.8 | 362 | 40.75 | Nucleus | 2540741 2545449 |
| *FvbZIP42* | LOC101313628 | 6 | A | 9.04 | 496 | 54.19 | Nucleus | 12611053 12614457 |
| *FvbZIP43* | LOC101299496 | 6 | UC | 7.59 | 531 | 57.95 | Nucleus | 12828095 12833118 |
| *FvbZIP44* | LOC101298723 | 6 | F | 6.5 | 255 | 28.39 | Nucleus | 14190196 14191881 |
| *FvbZIP45* | LOC101311204 | 6 | E | 6.19 | 329 | 37.04 | Nucleus | 15408471 15411641 |
| *FvbZIP46* | LOC101299607 | 6 | S | 8.91 | 229 | 26.35 | Nucleus | 20029544 20030829 |
| *FvbZIP47* | LOC101292813 | 6 | D | 7.79 | 451 | 50.04 | Nucleus | 22972602 22980557 |
| *FvbZIP48* | LOC101303726 | 6 | S | 5.88 | 219 | 25.03 | Nucleus | 30570778 30572044 |
| *FvbZIP49* | LOC101294103 | 7 | A | 8.4 | 320 | 35.72 | Nucleus | 355254 360820 |
| *FvbZIP50* | LOC101299415 | 7 | G | 9.28 | 427 | 45.95 | Nucleus | 4068927 4074293 |
| *FvbZIP51* | LOC101298745 | 7 | D | 8.42 | 334 | 37.2 | Nucleus | 9948832 9955917 |
| *FvbZIP52* | LOC101305009 | 7 | I | 6.3 | 364 | 40.1 | Nucleus | 19016616 19019686 |
| *FvbZIP53* | LOC101297896 | U | D | 7.18 | 536 | 60.55 | Nucleus | UC |
| *FvbZIP54* | LOC101305129 | U | I | 6.55 | 445 | 48.86 | Nucleus | UC |
