# Supplementary material for: Genome-Wide Identification and Expression Analysis of the Strawberry FvbZIP Gene Family and the Role of Key Gene FabZIP46 in Fruit Resistance to Gray Mold
Source: Plants (Basel). 2020 Sep 14;9(9):1199. doi: 10.3390/plants9091199 (PMC7569810; doi:10.3390/plants9091199)
Supplement: Supplementary file 1 [file plants-09-01199-s001.zip › plants-914991-SI/supplementary files/Table S3.docx]

**Table S3** Premier sequences used for qRT-PCR in this study.

| **Target** | **Forward sequence** | **Reverse sequence** |
| --- | --- | --- |
| FabZIP1 | TCTCATTCCCGTAGCCAAAAC | TCTCATTCCCGTAGCCAAAAC |
| FabZIP2 | AGGGTCTTCATCAGGTCATGG | GCTCGTAAGGATTTGGTTGTTCTC |
| FabZIP3 | CTGCATTTGGGAATGGACCT | CTGCCTACGGAACCTTGACC |
| FabZIP4 | GGCTTCAGCAGAGATGTTTCA | CAGTGAGATTCCTCCCACCTT |
| FabZIP5 | TTCCGTATCAGAAGCCAGTGAA | TCCCTCGTTGAAGGCAATAGA |
| FabZIP6 | CCTGGAAACCACTCAATTCCTC | TGCTTCACACGGATTTGCTC |
| FabZIP7 | CAATATGAGAGAAGGGATCAAAAGG | CAGGCGAAGGTGGTAGTTTAGG |
| FabZIP8 | CGTCTACAGCAACACCCTTCC | CATACCTCGTCCACCGTCTTC |
| FabZIP9 | GCTCTCTCTGATTGACCCCAAG | CACCTTCCTCTCCAGCTCATTT |
| FabZIP10 | GGAGAAAGAAGAGGCATTTGGA | ACACTTCTCAGCAACAAACCCTAAC |
| FabZIP11 | GGGGTTATGGGGAGTAGGATTT | CCTGTTGCTGTTGCTGACTTG |
| FabZIP12 | TGGACTGATGGAAACCCTGT | GCTCCTCTTGCTTGGTTATCTTG |
| FabZIP13 | CGATGGAGGATGTGTGGAAA | GAAAATGAAGCCACGAACACC |
| FabZIP14 | CCCTTCTCTCCCTAACCCTAAATC | CTCAACCAAATCAAACCACCA |
| FabZIP15 | GCATTCCAGCTCGAAGTTAAAGA | TCCCTGCCTGATACCGATG |
| FabZIP16 | CCTAGACCAGCTTCATCAGTCTCTC | TGGTGAAGGGTTTGCTGTCTC |
| FabZIP17 | GCTAAACCTCAACCATCACCATC | GACCCAAAACTCTTCCCACTCTT |
| FabZIP18 | CAATAAGTGGATGGATGAATGTTCC | TCATTTCACAATCACCACAGCA |
| FabZIP19 | AGCGAAAGCGGAAGAGAATG | TGCTGGGTGGTGATGTTTATG |
| FabZIP20 | AGCAACTCCAAAGATCCTAAATTCC | TTTTCCGTCTTGCGTTCTCTT |
| FabZIP21 | TGAAAAGGCACCACAGGAGAA | TCAGTTCCTTATTTCCACCCACA |
| FabZIP22 | TCGACCCTGGTTATATGGCTTT | CAGTTGCCGAATCCAATCCT |
| FabZIP23 | GCGTGTTCGGTGAGGTTATG | GTTGGGCAGAAAGAGTGGTTG |
| FabZIP24 | GCAGCAGACAAAACCCAAAG | AAGGTAAGTGAAGAGATTGGAGTGG |
| FabZIP25 | ACCCTCATCAACGAAAACACC | GGCCCTAATTCACAGTTTGTCC |
| FabZIP26 | AGGTTAAAGAGTGTTGCTGTCAAGG | AATCCTCCCAACCACATAAAACAC |
| FabZIP27 | GGACAACTTACCAAACTCCTTCCA | GACTGCATCGGAGTAGCGAAC |
| FabZIP28 | GAAGCTGAAGGGATTGGATGG | GGTGGTCTGCTGGAGTGTTTC |
| FabZIP29 | CATTGCTCACTAGATTGCCTTCTTT | TTTTCAATCCTACCTAATCCTCTGG |
| FabZIP30 | CGAGGCTTAACAGAGGAGGAAA | GAACAGAAACGACCACGGAAG |
| FabZIP31 | GCGGAACAAAGCCTTAACCA | GTAGAGCTGGTCCACTTCATGCT |
| FabZIP32 | TTTACAGGCAGAAGGGACTGAA | AATGCTCTTAGCCTCCCAATCTC |
| FabZIP33 | GATGGCGTCGTCGAAGTTAGT | GGTGATGTGGTGGTGCTTGT |
| FabZIP34 | GCTGCTATGCCGAGAACCA | TCCAGTAACCAAAGCAGGGAAC |
| FabZIP35 | AACGACAGCGGAGAAAGCA | GAGTGTGGCATTTTCATCTTTTAGG |
| FabZIP36 | ATCTGGGATTTCTAGGGGTTCTTT | TACAGGAAGGCGACGGAGA |
| FabZIP37 | CAACGACAGAGAAGCTCAGAAGAA | CCACGAATCAACGGCTAAGAA |
| FabZIP38 | TGCCAGAAATATCAGCAGCATC | ATCCTCTTCTGCCTCCTCTCAA |
| FabZIP39 | AACTTTCCTGGGTTTTGTATCTCTC | AACGTGAATCTTCAAAGCATAGACC |
| FabZIP40 | GCTGGGAATGATTTATGCAGGT | CACTTGTTGCTTGCCTTATTGG |
| FabZIP41 | ATGGGACTTGGTACAGGAGACATAC | CAGTTGGAGCAAGACTGGTTATTTT |
| FabZIP42 | CAGCAATGTCCAGCACCAC | CCAAACTTCATCCACTGTCTTCC |
| FabZIP43 | TTGACCAACTCAACTCCTATCTGC | CTGCGTATTCTCCGTTCTTCTTTT |
| FabZIP44 | ATTGATAGTGAGTTGGGTGCTTTTC | GCAGTTCACAATCCCCTTCC |
| FabZIP45 | TCAGCGACTCCATCACATTTCT | CTCATCGTCAAACCTGTCAAACTC |
| FabZIP46 | GATCAAGAGAATGATTTCCAACAGG | GAGAGCTGACGATTTGTGATGTG |
| FabZIP47 | GTTTTATGTGGATTGGTGGCTTC | CTGGGATGATTGCTGTAAGTTGT |
| FabZIP48 | AATTCCCTTCCCACTTAGTTCCTC | CATTCGTCTCTGTTTCCTCTCGT |
| FabZIP49 | TGTAGGGGCTGGTGCTGTAA | CATTCTCTTCTGCCTCCTCTCAA |
| FabZIP50 | ATGTTGAGAATGCTGAAGGTGAAG | GGATTGATTTGCCCGAGAAG |
| FabZIP51 | TGAAGGGCACAGCAGCTAAA | CAGACGAACGAAAACCACCA |
| FabZIP52 | ATGGACGGGTCGTTGGAAT | AAAATCCTCTTGGCTCGCTTG |
| FabZIP53 | ACTGACGGAGCAGCAACTAATG | GGCTATGGTGTCCGAGAGAGA |
| FabZIP54 | CAAACGATGGAGCAACAGG | CAGCACCAAAAGAAGCGAAG |
| FaPR1 | CTAAATATCTTCTTCCTGCCATA | AATGTTGTATCTATTGCTGTT |
| FaPR4 | TCGTGTTCTTGGTGATGTTGTG | TGCGGGTTGTAGAGGTGGT |
| FaWRKY1 | TGTGCAGGGTGTGCTCTCTT | TGGGATTTGGATTGCCTTTTAC |
| FaWRKY33 | ACCGTTCAACTGAGCAACAAGA | AAGAAGGGGAAGGAGGAGGAG |
| FaWRKY70 | GGGCGTCAAGGAAGAAGAGA | CGGACGACTCAAGCACACA |
| FaPGIP1 | TGCTAGAATTCGATCTGTCCAAGG | ATTATCCAATTGGGTCAACTGCTC |
| FaPGIP2 | TCCTCATGGAAATCCGACGCCGAC | ACCTGTGAGATTGGGGAGCTTGCG |
| FaCHI2 | GCACAACAGGTGATGTTGC | GTAATGACGTCGTGGCTTGA |
| FaCHI3 | AGGTCTTCTTAGGACTCCCTG | CTTGGACCAAAGCATGACACCGCC |
| FaCHI4 | AGCGAAAGCCATTGAAAAGT | CATTTGGTGATTGTGTGAAGAG |
| FaActin | GCCAACCGTGAGAAGATG | TCCAGAGTCAAGAACAATACCAG |
